# Complete chloroplast and mitochondrial genomes of *Ditrichum rhynchostegium* Kindb. (Ditrichaceae, Bryophyta)

**DOI:** 10.1080/23802359.2023.2185465

**Published:** 2023-03-08

**Authors:** Yuya Inoue, Miho Nakahara-Tsubota, Eri Ogiso-Tanaka, Hiromi Tsubota

**Affiliations:** a Department of Botany, National Museum of Nature and Science, Ibaraki, Japan; b Hattori Botanical Laboratory, Miyazaki, Japan; c Cooperative Research Fellow of Natural History Museum and Institute, Chiba, Japan; d Center for Molecular Biodiversity Research, National Museum of Nature and Science, Ibaraki, Japan; e Miyajima Natural Botanical Garden, Graduate School of Integrated Sciences for Life, Hiroshima University, Hiroshima, Japan

**Keywords:** Bryophyta, Ditrichaceae, chloroplast genome, mitochondrial genome, phylogenetic relationships

## Abstract

The moss family Pottiaceae is one of the most diverse lineages of the subclass Dicranidae (haplolepideous mosses). Nevertheless, the phylogenetic relationships of Pottiaceae with other Dicranidae families remain unclear. To better understand the ancestral genomic structure and evolution of the Pottiaceae, herein, we present the chloroplast and mitochondrial genomes of *Ditrichum rhynchostegium* (Ditrichaceae, Bryophyta). The chloroplast genome is 124,628 bp long and displayed a circular structure composed of a large single-copy region, a small single-copy region, and a pair of inverted repeats. It has 118 genes, including 82 protein-coding genes, 32 tRNA genes, and four rRNA genes. The mitochondrial genome is 106,246 bp long and has a circular structure. It contains 67 genes, including 40 protein-coding genes, 24 tRNA genes, and three rRNA genes. Phylogenetic trees based on the coding sequences strongly support the sister relationship of *D. rhynchostegium* with all Pottiaceous accessions, and the dextrosely arranged operculum cells suggest its affinity for Pottiaceae. This study also demonstrates that long-read sequencing employing the Nanopore platform facilitates the repair of unassembled or misassembled organellar genomic regions.

## Introduction

The family Pottiaceae is the most species-rich lineage of mosses, with more than 1400 widely distributed species across approximately 80 genera (Zander 1993). Globally, this species has adapted to a broad variety of habitat types, including xeric, mesic, and hydric habitats, growing on a variety of substrata, presenting saxicolous, terricolous, and corticolous natures, and employing various life strategies, including perennial, annual, and ephemeral (Inoue and Tsubota 2016). Several species in this family exhibit numerous adaptations to harsh environments, which are referred to as the ‘Xeropottioid life syndrome’ (Frey and Kürschner 1988). This family is one of the most diverse lineages of the Dicranidae subclass (haplolepideous mosses). Nevertheless, the phylogenetic relationships of Pottiaceae with other Dicranidae families remain unclear. Reconstructing a robust phylogeny based on genomic data could provide a better understanding of the evolution and diversification of Pottiaceae.

Many Pottiaceae genera have provided genomic resources for organelles (*Chionoloma*: Alonso et al. 2016, as *Oxystegus*; *Pseudocrossidium*: Cevallos et al. 2019, 2020; *Syntrichia*: Oliver et al. 2010; Yoon et al. 2016; Kim et al. 2019; *Scopelophila*: Inoue et al. 2022). However, genomic data for other Dicranidae families are still scarce, and no organelle genome data exist for the family Ditrichaceae *s. str*., which appears to be the closest relative to Pottiaceae (Stech et al. 2012; Inoue and Tsubota 2014; Fedosov et al. 2016).

To better understand the ancestral genomic structure and evolution of the family Pottiaceae, we present the chloroplast and mitochondrial genomes of *Ditrichum rhynchostegium* Kindberg 1910 (Ditrichaceae). The protein-coding sequences of the chloroplast and mitochondrial genomes were used to estimate the phylogenetic relationship between *D. rhynchostegium* and the Pottiaceae clade.

## Materials and methods

**Figure 1.**
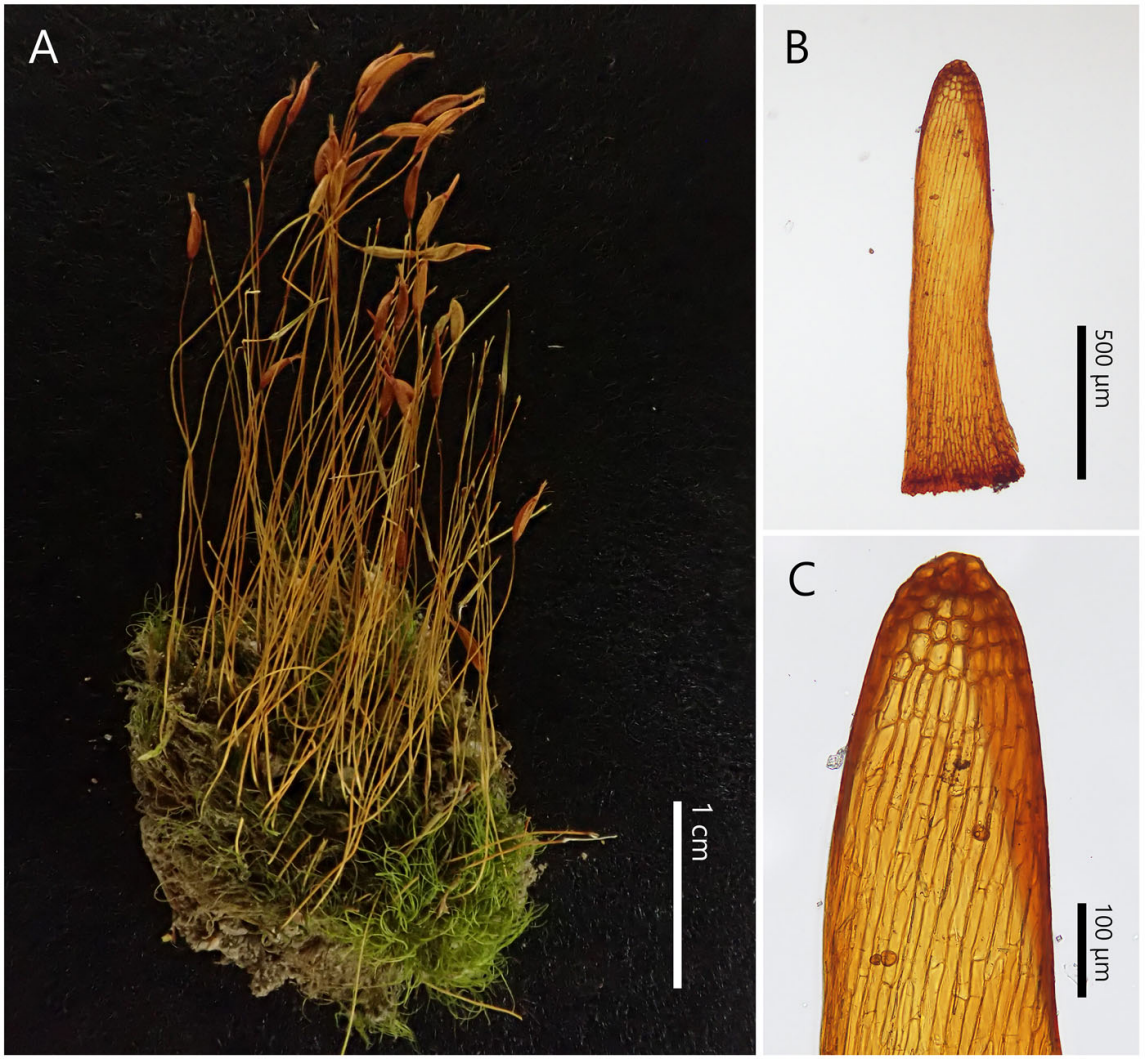
*Ditrichum rhynchostegium*. (A) Dry habit. (B) Operculum (outer surface view). (C) Close-up view of the upper part of (B). All photographs were taken from the voucher specimen *Y. Inoue 5462* (HIRO).

*Ditrichum rhynchostegium* samples (Figure 1) were collected from Yamaguchi Prefecture, Japan (34°00′24″N, 131°22′29″E). The voucher specimen was deposited at the Herbarium of Hiroshima University (HIRO: http://sweetgum.nybg.org/science/ih/herbarium-details/?irn=124749, Prof. Tomio Yamaguchi, yamatom@hiroshima-u.ac.jp) under collector number *Y. Inoue 5462*. Total DNA was extracted from the axenic *in vitro* culture of protonemal filaments that are derived from a single spore (Takio 1995, modified), with NucleoSpin Plant II (Macherey-Nagel, Duren, Germany) following the manufacturer’s protocols. Library preparation and sequencing were performed by Genome-Lead, Ltd. (Takamatsu, Japan) using the MGI DNBSEQ-G400RS FAST platform. Approximately, 42.57 M raw reads were analyzed, comprising an average fragment length of 150 bp. Low-quality reads (<Q30), abnormal short reads (<20 bp), and adapter sequences were trimmed using Fastp ver. 0.20.1 (Chen et al. 2018).

After quality control, GetOrganelle ver. 1.7.1 (Jin et al. 2020) was used for *de novo* assembly, and the assembled sequences were polished using Pilon ver. 1.23 (Walker et al. 2014). The polished sequences were annotated using GeSeq ver. 2.03 (Tillich et al. 2017) and manually corrected using SnapGene ver. 5.3.2 (GSL Biotech, https://www.snapgene.com). To verify the accuracy of the assembly, we mapped clean reads to the assembled chloroplast and mitochondrial genomes to assess the depth of coverage (Figs. S1 and S2).

Figure 2.Circular maps of *Ditrichum rhynchostegium* chloroplast (A) and mitochondrial (B) genomes. In the chloroplast genome, the small single-copy (SSC) and large single-copy (LSC) regions are separated by inverted repeats (IRs, IRA, and IRB). Genes inside the map are transcribed clockwise, and genes outside are transcribed counterclockwise. Genes with related functions are shown in the same color. Asterisks denote genes containing introns.

**Figure:**
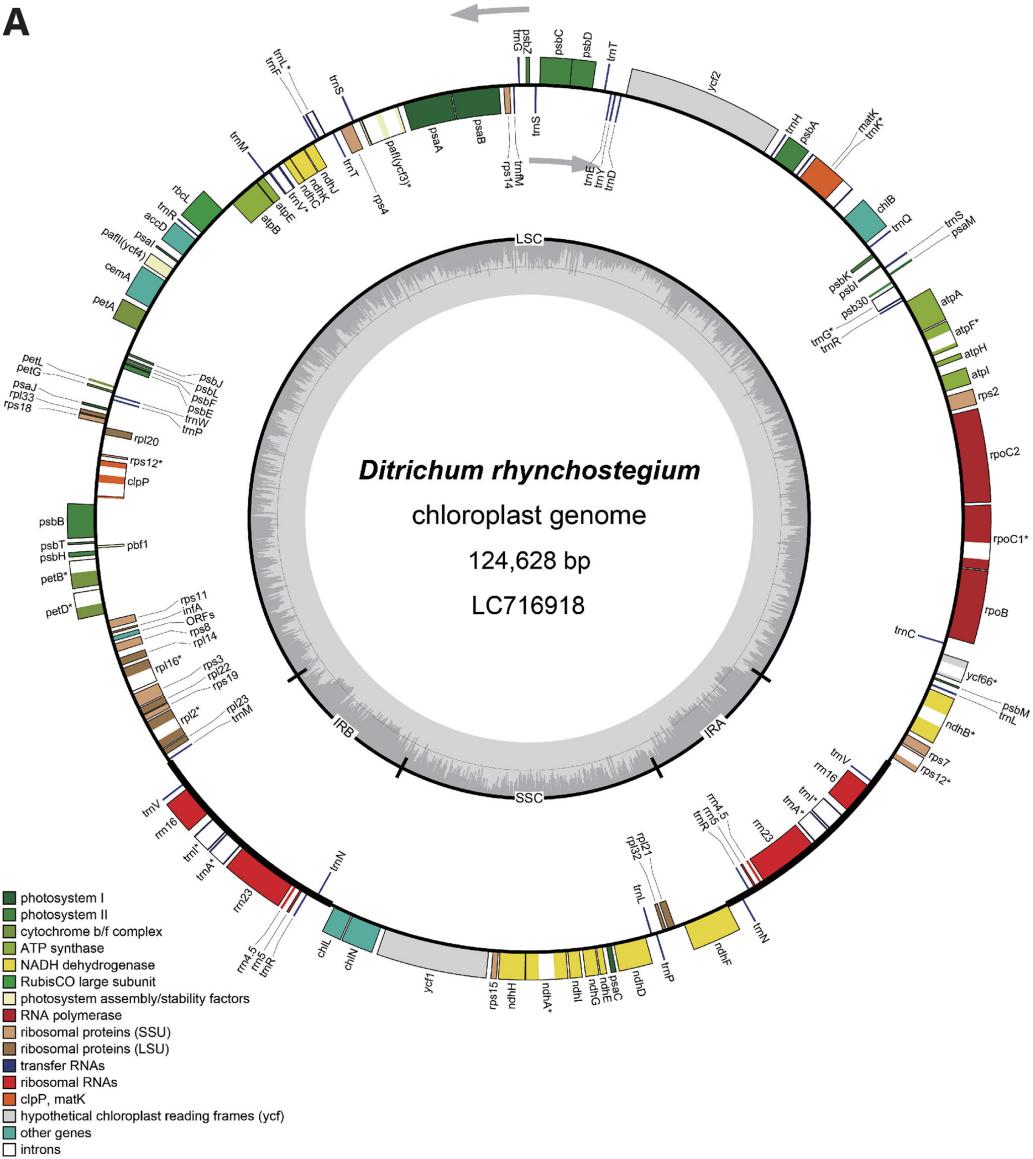

**Figure:**
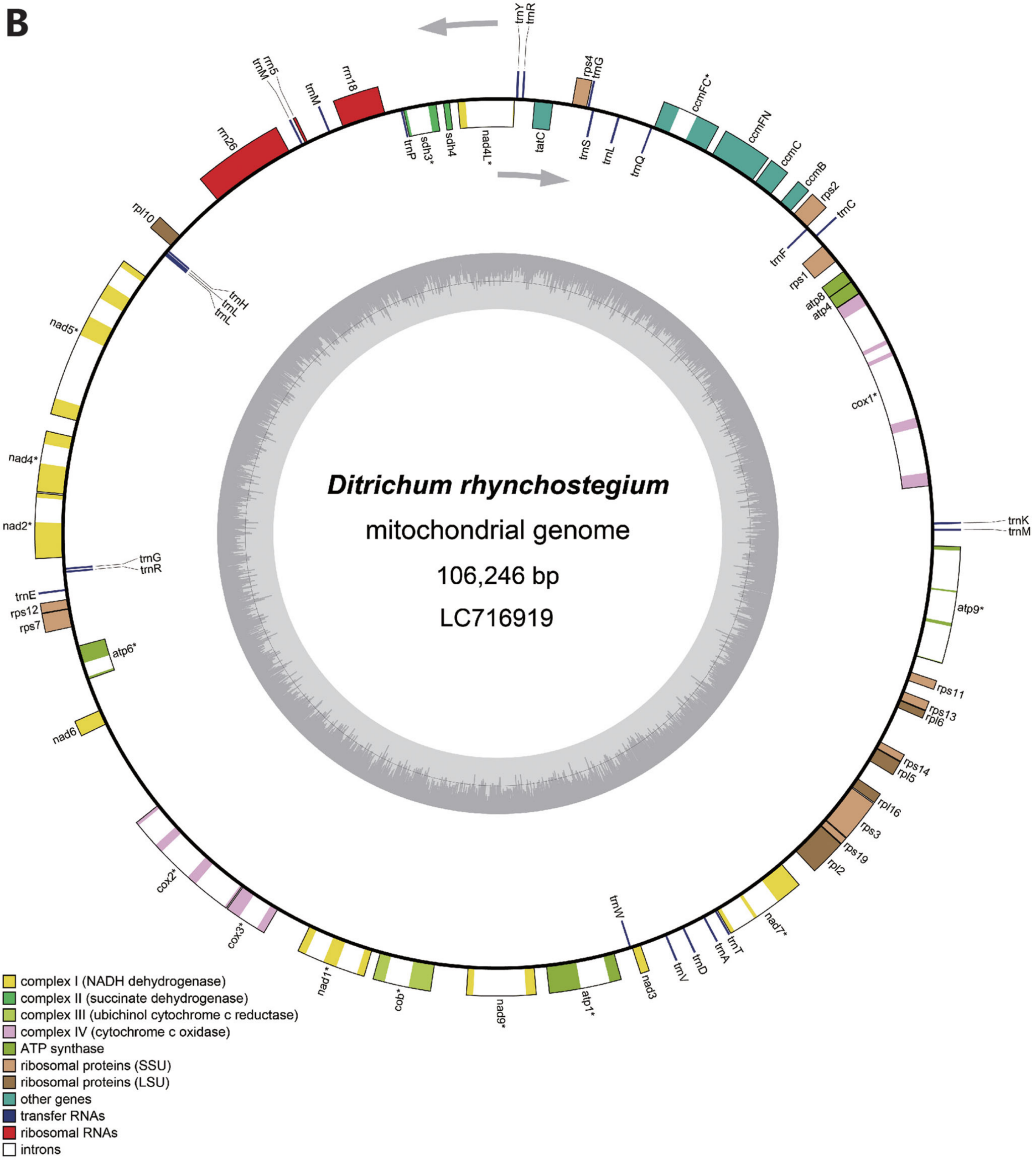

The assembled mitochondrial genome contained gaps; thus, specific primers were designed to bridge these gaps (Table S1). The PCR conditions were as described by Inoue and Aung (2021). The DNA library was prepared from the amplicons (100–200 ng) using a Ligation Sequence Kit (SQK-LSK109; Oxford Nanopore Technologies (ONT), Oxford, UK) after phosphorylation of the PCR products (Ogiso-Tanaka et al. 2021). Amplicon sequencing was performed using MinION or Flongle flow cells (R9.4; ONT). Guppy GPU v0.5.0 R9.4.1, with the HAC model (ONT) was used for basecalling. The basecalled reads were BLASTed locally against the target regions of the assembled sequence using BLASTN, and the BLAST hit sequence was filtered by identity (>90%) and read length of PCR product size. Consensus sequences were obtained from the filtered reads using NGSpeciesID software (Sahlin et al. 2021).

Circular maps were generated from the final annotated chloroplast and mitochondrial sequences using OGDRAW ver. 1.3.1 (Greiner et al. 2019). The structures of intron-containing genes were also visualized (Figs. S3 and S4). The chloroplast and mitochondrial sequences were submitted to the DNA Data Bank of Japan (DDBJ), and accession numbers LC716918 and LC716919 were assigned to the chloroplast and mitochondrial genomes, respectively.

**Figure 3.**
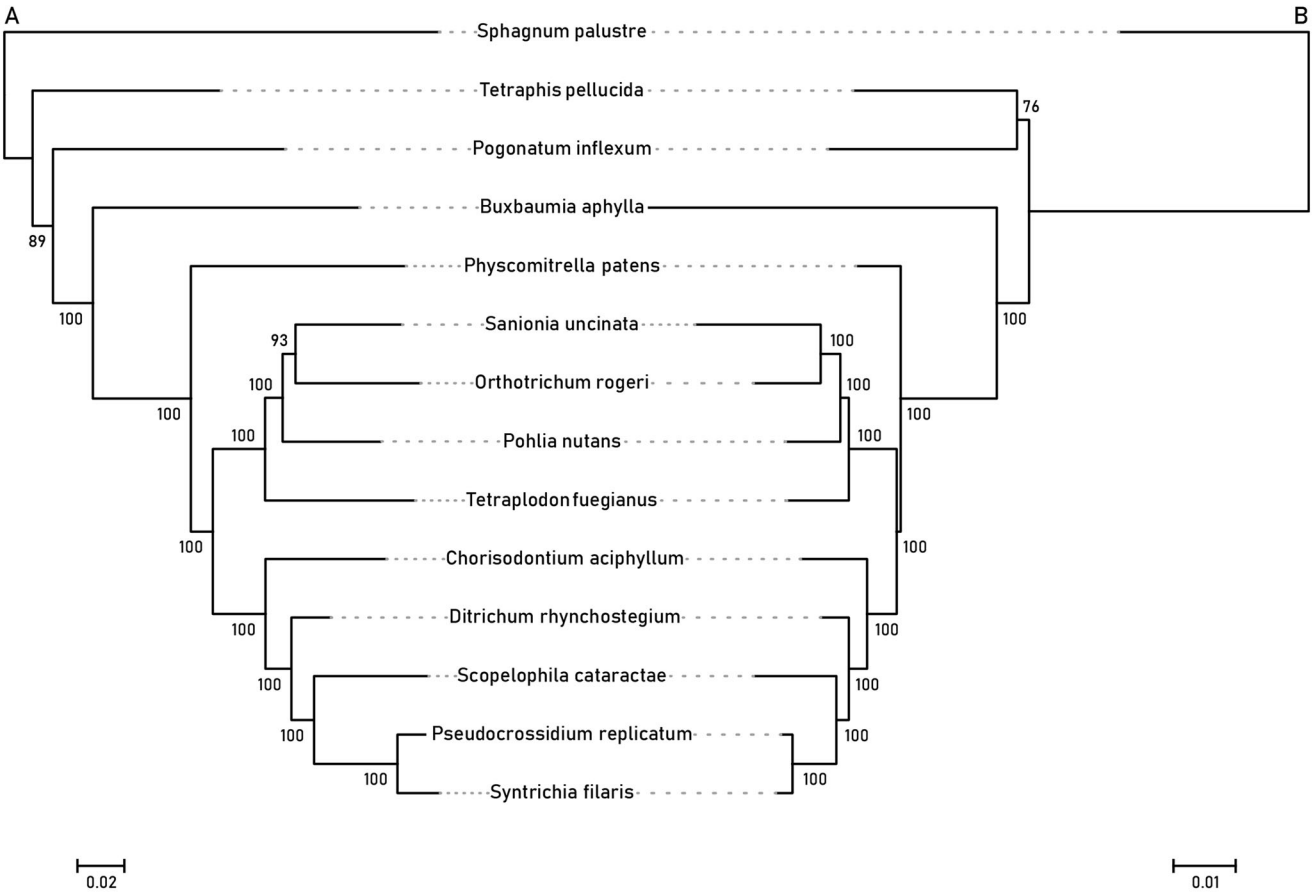
Maximum-likelihood trees of mosses inferred from the (A) 80 chloroplast and (B) 39 mitochondrial protein-coding sequences. Bootstrap values of 10,000 replicates by RAxML are shown on the branches. The root is arbitrarily placed on the branch leading to *Sphagnum palustre.*

Phylogenetic analyses were conducted using protein-coding sequences of the chloroplast (80 genes) and mitochondrial (39 genes) genomes. Each data matrix consisted of representative species selected from the major lineages of mosses (Table S2), based on the study by Liu et al. (2019). Sequences were aligned using MAFFT ver. 7.515 (Katoh and Standley 2013), with few manual adjustments using the sequence editor of MEGA ver. 7.0.26 (Kumar et al. 2016). Start and stop codons were removed, and gaps were treated as missing data. Kakusan4 (ver. 4.0.2016.11.07; Tanabe 2011) was used to determine the substitution model and partitioning scheme based on the corrected Akaike information criterion (AICc; Sugiura 1978). RAxML ver. 8.2.12 (Stamatakis 2014) was used for maximum-likelihood inference using the GTR + Γ model with a rapid bootstrap analysis of 10,000 replicates.

## Results and discussion

The chloroplast genome of *D. rhynchostegium* is a 124,628-bp circular DNA molecule with a typical quadripartite structure composed of a large single-copy (LSC) region of 85,897 bp, a small single-copy (SSC) region of 18,753 bp, and a pair of inverted repeats (IRs) of 9989 bp (Figure 2(A)). The genome’s GC content is 29%. There were 118 genes in the chloroplast genome, including 82 protein-coding genes, 32 tRNA genes, and four rRNA genes. Similar to *Scopelophila cataractae* (Inoue et al. 2022), the chloroplast genome of *D. rhynchostegium* contains *trnP*^GGG^ as a functional gene, which is absent in other published chloroplast genomes of Pottiaceae (*Pseudocrossidium*: Cevallos et al. 2019; *Syntrichia*: Oliver et al. 2010; Kim et al. 2019). The mitochondrial genome is a 106,246-bp circular DNA molecule (Figure 2(B)). The genome’s GC content is 40%. There are 67 genes in the mitochondrial genome, including 40 protein-coding genes, 24 tRNAs, and three rRNAs. Although the mitochondrial genome contained several gaps following short read assembly, amplicons sequenced using the Nanopore platform bridged these gaps. This suggests that, similar to traditional Sanger sequencing, the Nanopore platform is a viable option for sequencing amplicons.

The chloroplast genome data matrix was 64,895 bp long, with 25,108 variable sites (39%) and 12,481 parsimony-informative sites (50% of the variable sites). The mitochondrial genome data matrix was 30,066 bp, with 6163 variable sites (20%) and 2267 parsimony-informative sites (37% of the variable sites). Figure 3 depicts the maximum-likelihood tree of the representative mosses, including the Pottiaceous accessions, with bootstrap support values. Despite the differences in backbone topology, particularly in the relationship between Tetraphidopsida and Polytrichopsida, *Ditrichum rhynchostegium* was resolved as a sister to the Pottiaceae clade in both the chloroplast and mitochondrial trees with high supporting values (Figure 3). The closest relationship between Ditrichaceae *s. str.* with Pottiaceae was consistent with the findings of phylogenetic studies based on selected chloroplast and mitochondrial markers (Inoue and Tsubota 2014; Fedosov et al. 2016). The dextrosely arranged operculum cells of *D. rhynchostegium* (Figure 1(B,C)) also suggested a close relationship with Pottiaceae, whose opercula are often composed of dextrosely arranged cells (Zander 1993).

## Supplementary Material

Supplemental Material

Supplemental Material

Supplemental Material

Supplemental Material

Supplemental Material

## Data Availability

The genome sequence data that support the findings of this study are openly available at NCBI (https://www.ncbi.nlm.nih.gov/) under the accession numbers LC716918 and LC716919. The associated BioProject, BioSample, and SRA numbers are PRJDB11883 and SAMD00495089, DRX365852, and DRX400239, respectively.
