# Supplementary material for: Complete chloroplast and mitochondrial genomes of Ditrichum rhynchostegium Kindb. (Ditrichaceae, Bryophyta)
Source: Mitochondrial DNA B Resour. 2023 Mar 8;8(3):383–8. doi: 10.1080/23802359.2023.2185465 (PMC10013369; doi:10.1080/23802359.2023.2185465)
Supplement: Supplemental Material [file TMDN_A_2185465_SM9537.pdf]

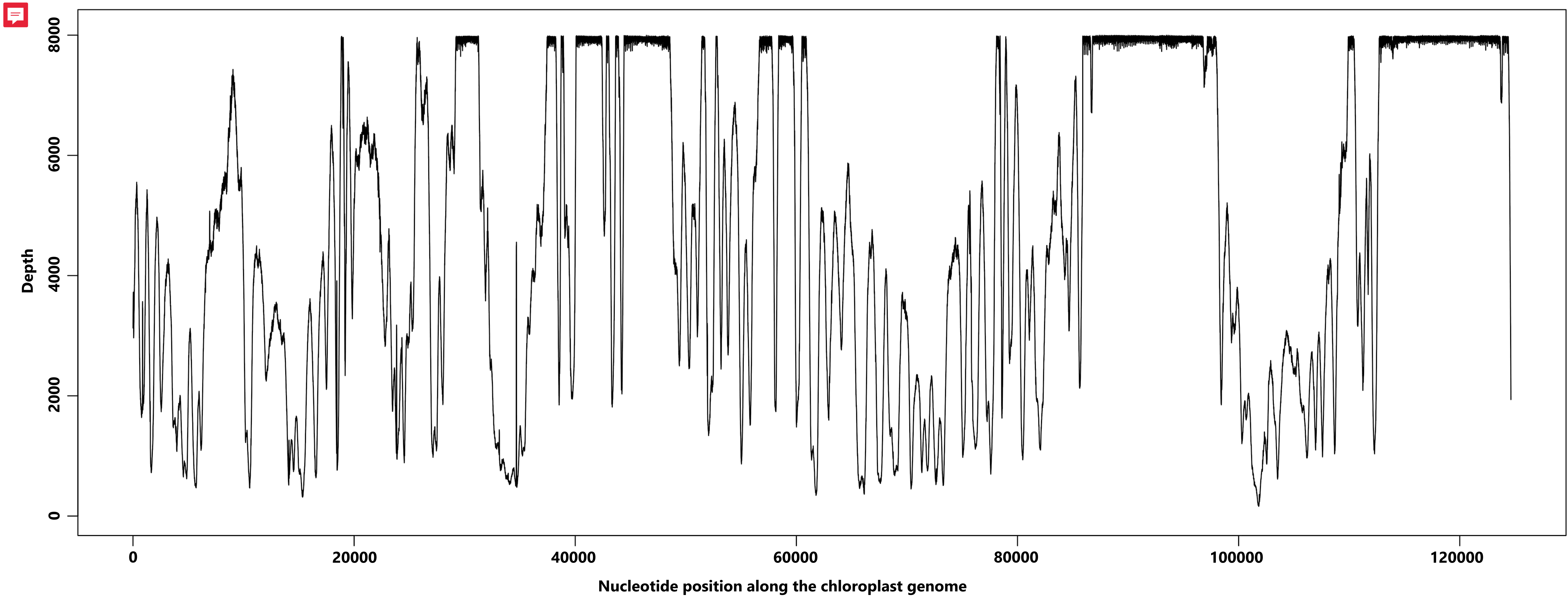

Depth
8000
6000
4000
2000
0
0
20000
40000
60000
80000
100000
120000
Nucleotide position along the chloroplast genome

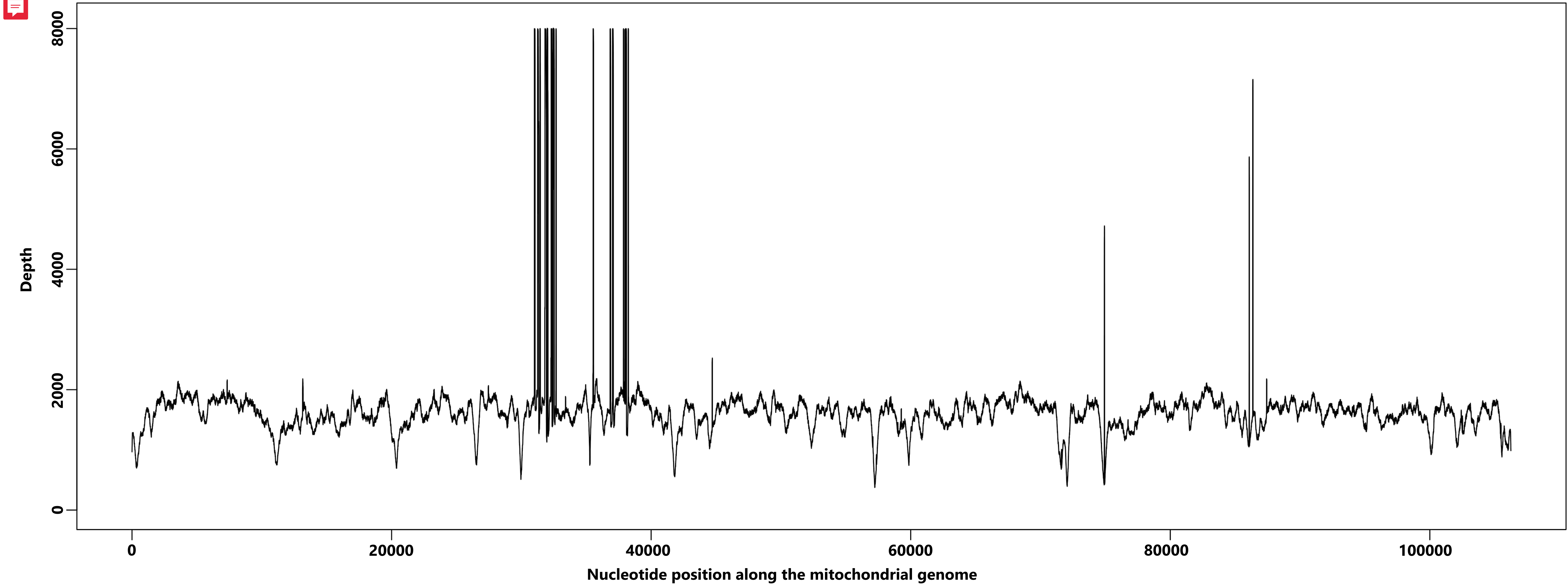

Depth
8000
6000
4000
2000
0
0
20000
40000
60000
80000
100000
Nucleotide position along the mitochondrial genome

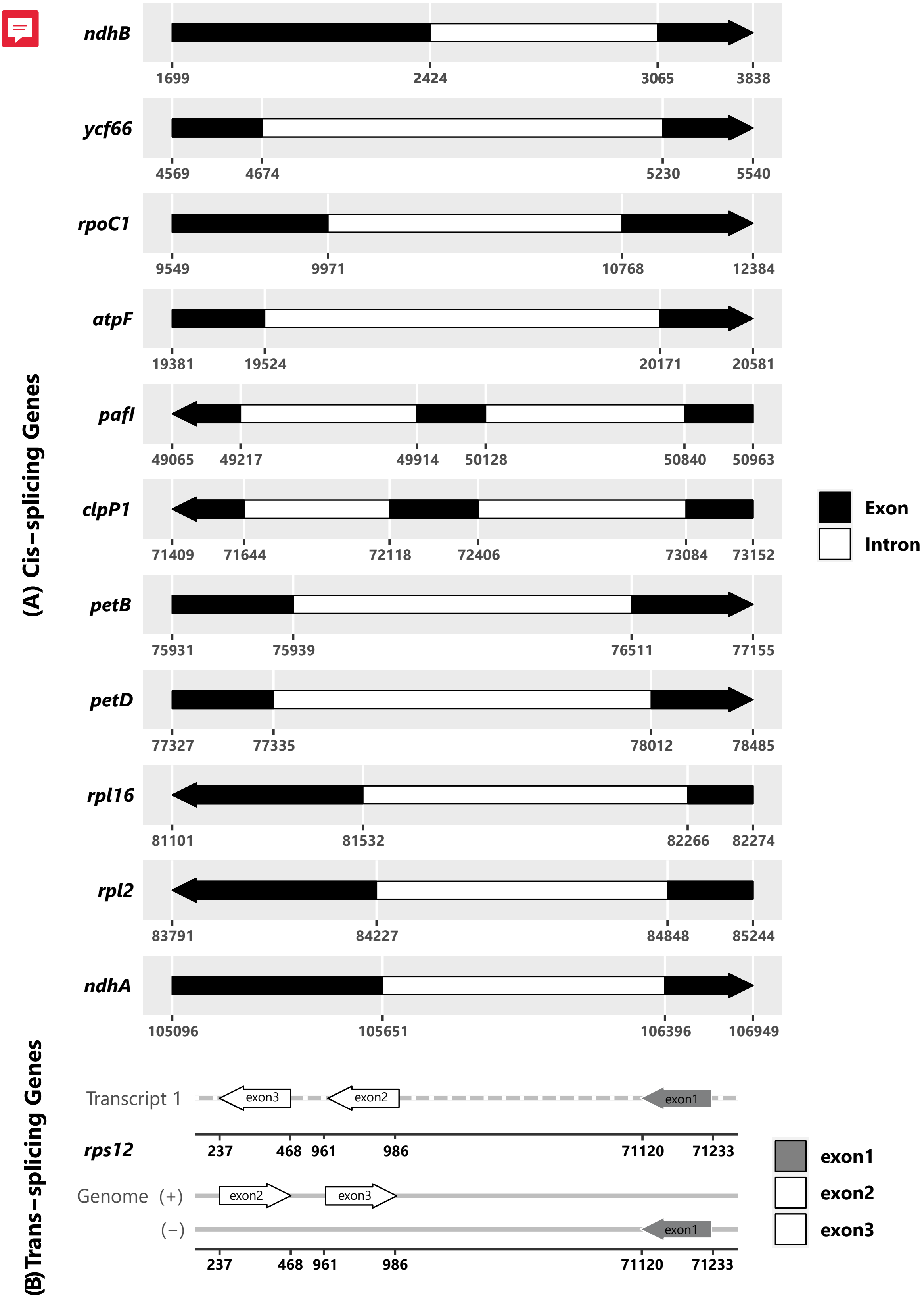

(A) Cis−splicing Genes
ndhB
1699
2424
3065
3838
ycf66
4569
4674
5230
5540
rpoC1
9549
9971
10768
12384
atpF
19381
19524
20171
20581
pafI
49065
49217
49914
50128
50840
50963
clpP1
71409
71644
72118
72406
73084
73152
petB
75931
75939
76511
77155
petD
77327
77335
78012
78485
rpl16
81101
81532
82266
82274
rpl2
83791
84227
84848
85244
ndhA
105096
105651
106396
106949
Exon
Intron
(B) Trans−splicing Genes
Transcript 1
exon3
exon2
exon1
rps12
237
468
961
986
71120
71233
Genome (+)
exon2
exon3
(−)
exon1
237
468
961
986
71120
71233
exon1
exon2
exon3

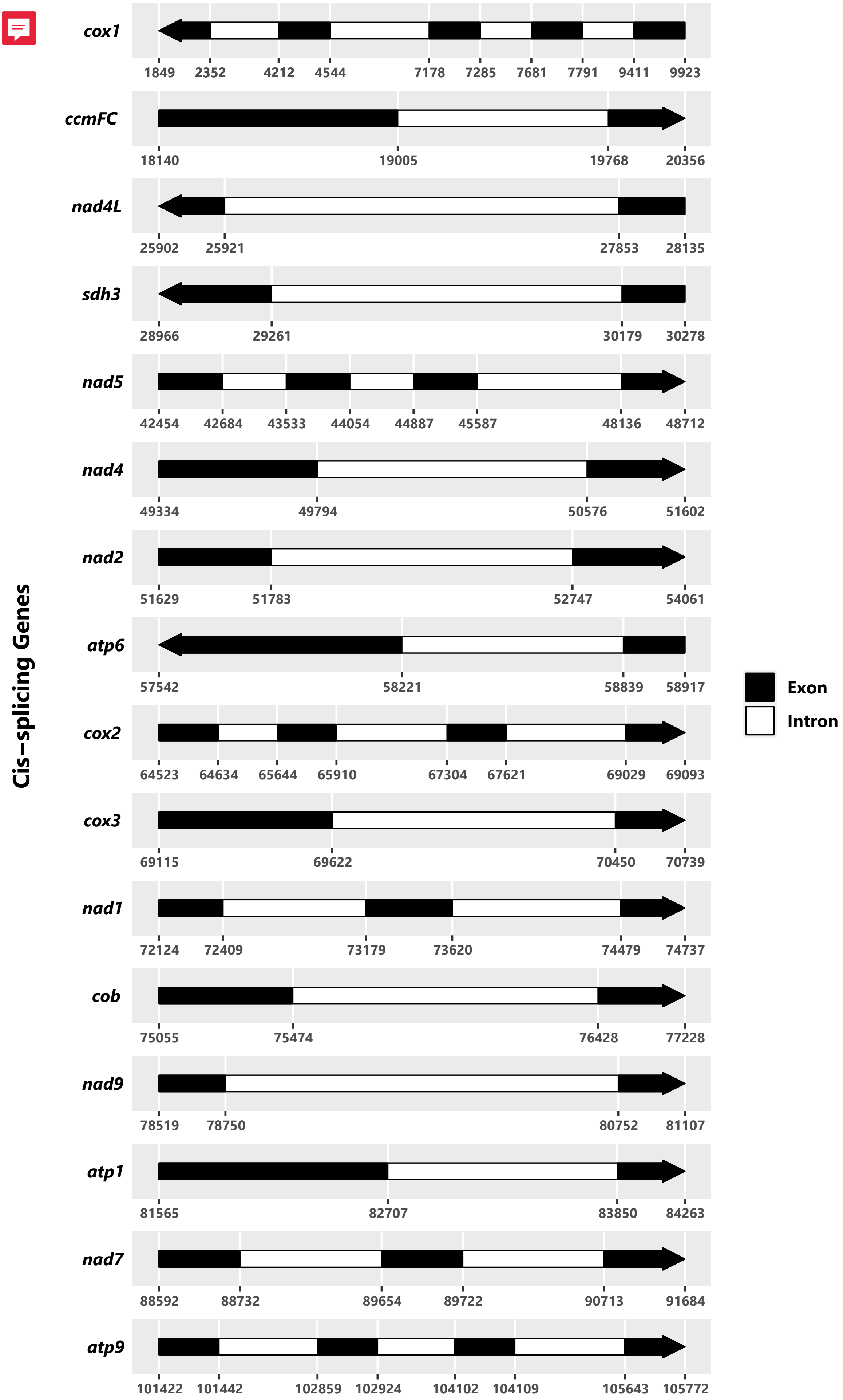

Cis−splicing Genes
cox1
1849 2352 4212 4544 7178 7285 7681 7791 9411 9923
ccmFC
18140 19005 19768 20356
nad4L
25902 25921 27853 28135
sdh3
28966 29261 30179 30278
nad5
42454 42684 43533 44054 44887 45587 48136 48712
nad4
49334 49794 50576 51602
nad2
51629 51783 52747 54061
atp6
57542 58221 58839 58917
cox2
64523 64634 65644 65910 67304 67621 69029 69093
cox3
69115 69622 70450 70739
nad1
72124 72409 73179 73620 74479 74737
cob
75055 75474 76428 77228
nad9
78519 78750 80752 81107
atp1
81565 82707 83850 84263
nad7
88592 88732 89654 89722 90713 91684
atp9
101422 101442 102859 102924 104102 104109 105643 105772
Exon
Intron

Primer name	Sequence (5’-3’)	Target region	Note
YI5462a5Nmt1	AAGTCCTCGCAAGCTTTCCC	*nad9*	Designed at *cob* gene
YI5462a5Nmt2	AGTTCTGCTCCAGCCAACTTA	*nad9*	Designed at *atp1* gene
YI5462a5Nmt3	AGGGAAAGGTCTCCGGTTCA	*nad7*	Designed at *trnT*^{GGU} gene
YI5462a5Nmt4	ACGGTAATACGCCCTGATGA	*nad7*	Designed at *rpl2* gene
YI5462a5Nmt5	CTACCAGACTGCGCTACACC	*rrn18*	Designed at *trnP*^{UGG} gene
YI5462a5Nmt6	ATCCCGCTGTTCTAACCGAC	*rrn18*	Designed at *trnM*^{CAU} gene

Class	Subclass	Order	Family	Species	Accession number	
					Chloroplast	Mitochondrion
Sphagnopsida		Sphagnales	Sphagnaceae	Sphagnum palustre	KU726621	KC784957
Tetraphidopsida		Tetraphidales	Tetraphidaceae	Tetraphis pellucida	KJ817846	KJ817845
Polytrichopsida		Polytrichales	Polytrichaceae	Pogonatum inflexum	MK131349	MK131350
Bryopsida	Buxbaumiidae	Buxbaumiales	Buxbaumiaceae	Buxbaumia aphylla	MN496310	KC784954
	Funariidae	Funariales	Funariaceae	Physcomitrium patens	AP005672	KY126309
	Dicranidae	Dicranales	Dicranaceae	Chorisodontium aciphyllum	MW355440	MK651511
			Ditrichaceae	Ditrichum rhynchostegium	**LC716918**	**LC716919**
		Pottiales	Pottiaceae	Pseudocrossidium replicatum	MG132071	MT310681
				Scopelophila cataractae	LC634773	LC634774
				Syntrichia filaris	MK852705	KP984758
	Bryidae	Splachnales	Splachnaceae	Tetraplodon fuegianus	KU095851	KT373818
		Bryales	Mniaceae	Pohlia nutans	MN937553	MN956803
		Orthotrichales	Orthotrichaceae	Orthotrichum rogeri	KP119739	KM873610
		Scorpidiaceae	Hypnales	Sanionia uncinata	KM111545	KP984757
