# Supplementary figures and images for: Complete chloroplast and mitochondrial genomes of Ditrichum rhynchostegium Kindb. (Ditrichaceae, Bryophyta)
Source: Mitochondrial DNA B Resour. 2023 Mar 8;8(3):383–8. doi: 10.1080/23802359.2023.2185465 (PMC10013369; doi:10.1080/23802359.2023.2185465)

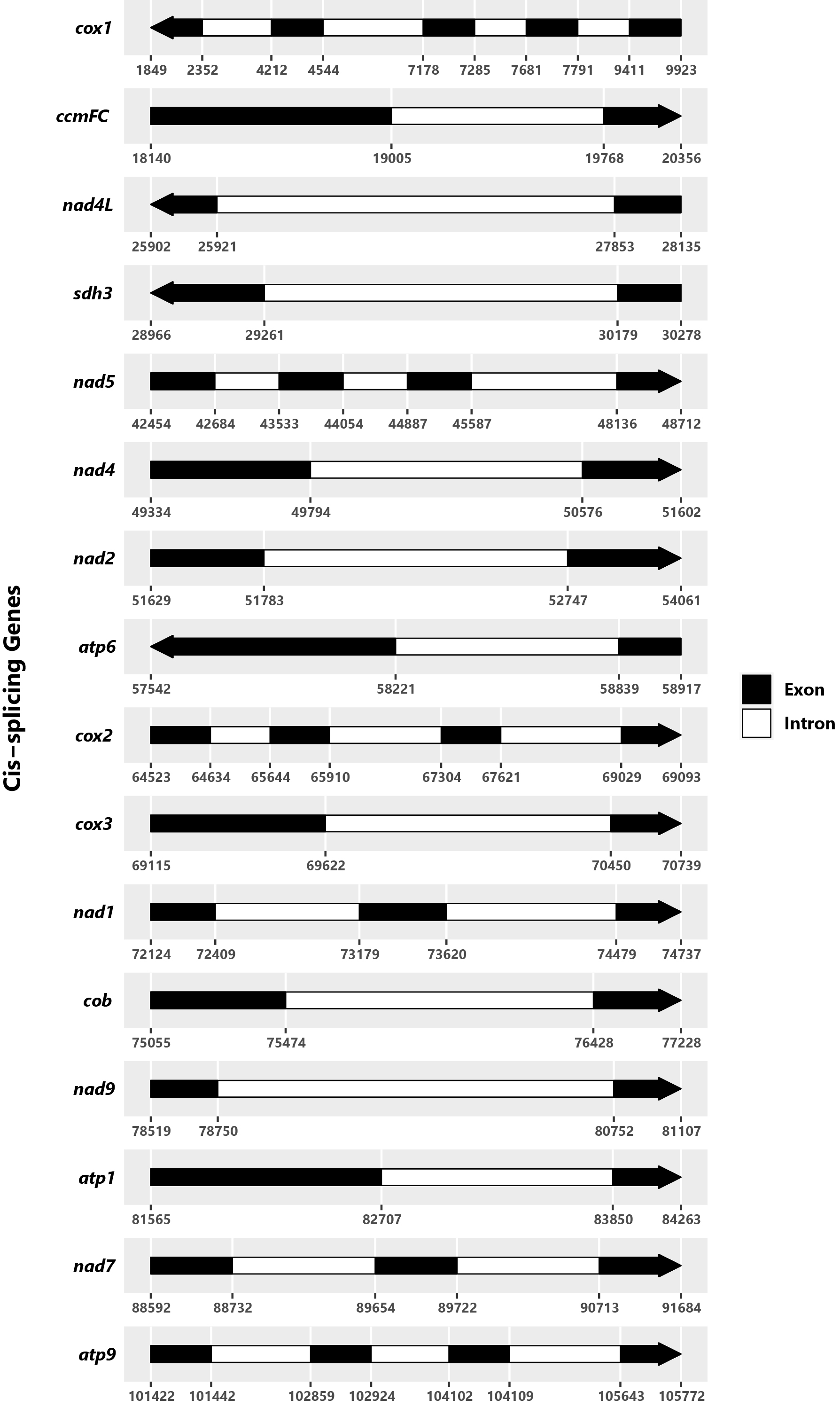

Supplement: Supplemental Material [file TMDN_A_2185465_SM9532.jpg]

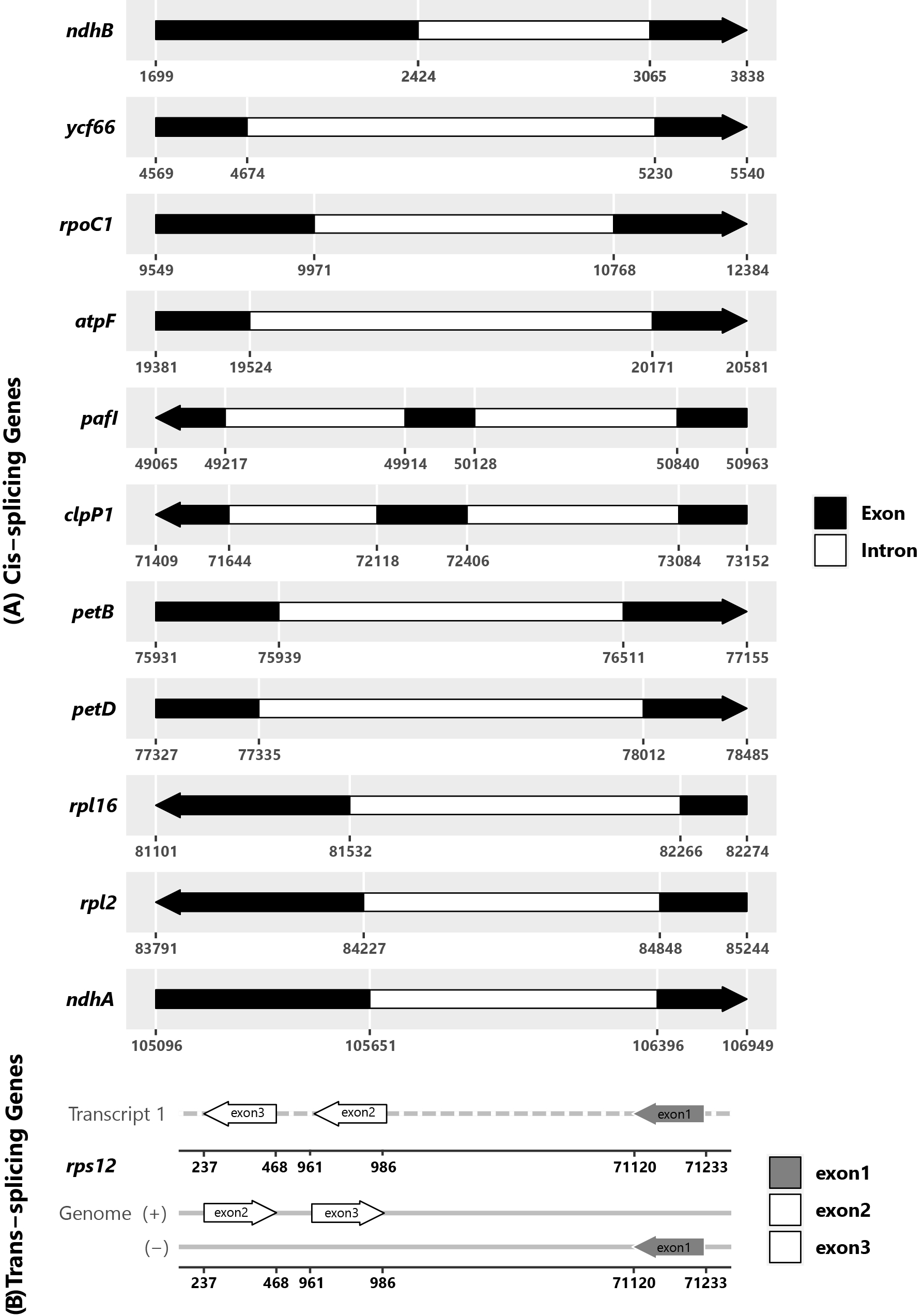

Supplement: Supplemental Material [file TMDN_A_2185465_SM9525.jpg]

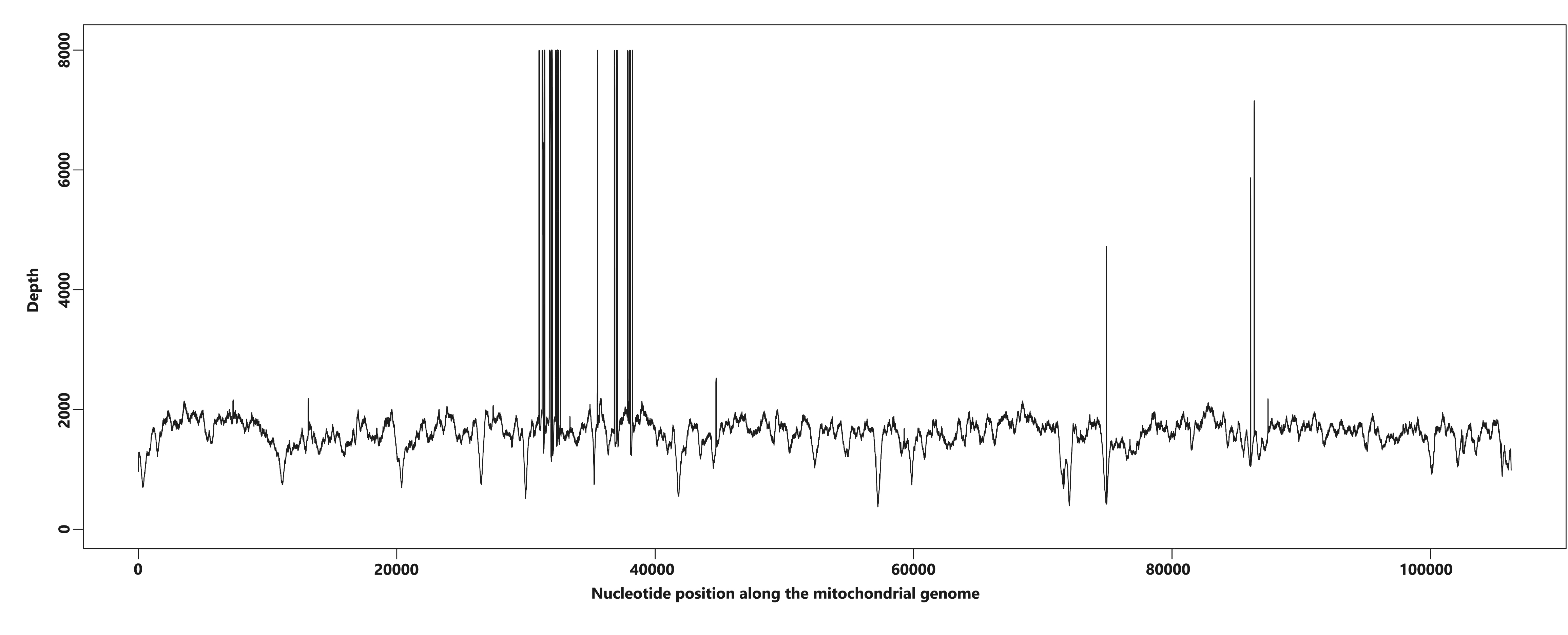

Supplement: Supplemental Material [file TMDN_A_2185465_SM9490.jpg]

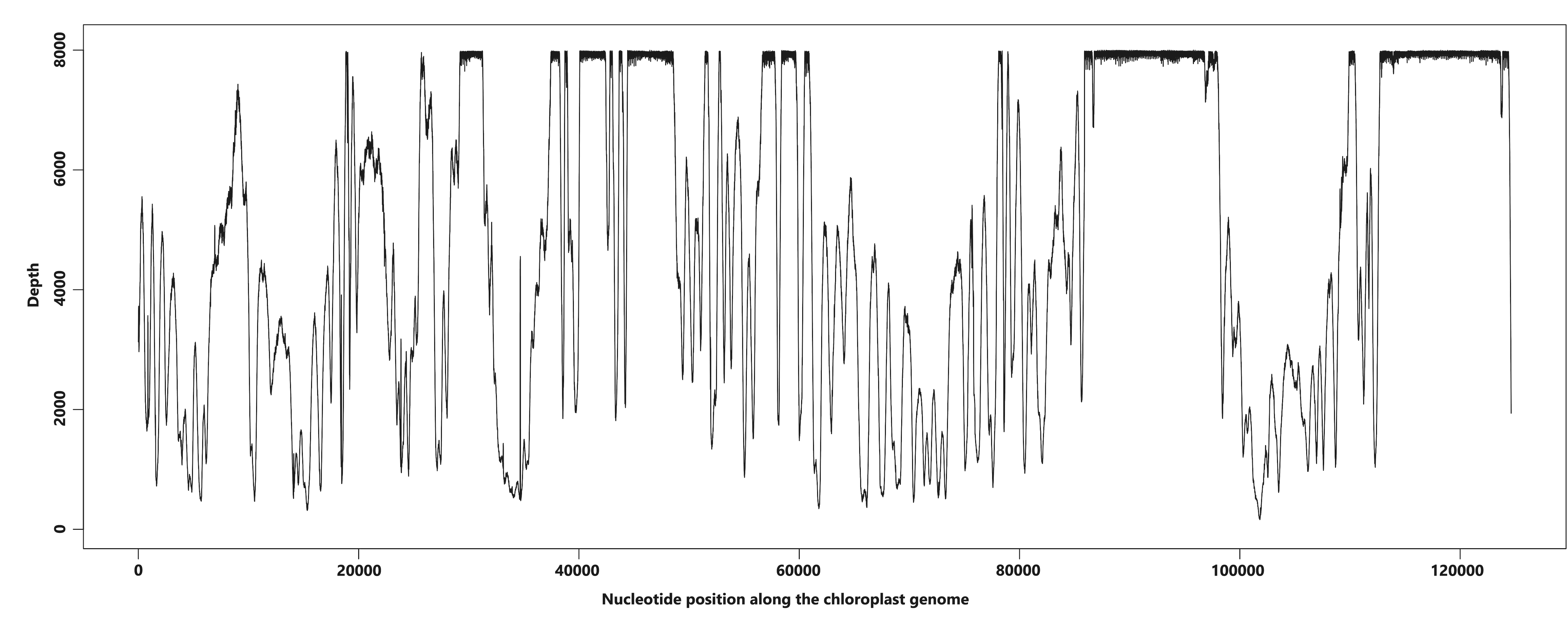

Supplement: Supplemental Material [file TMDN_A_2185465_SM9489.jpg]
